# NLRP12 collaborates with NLRP3 and NLRC4 to promote pyroptosis inducing ganglion cell death of acute glaucoma

**DOI:** 10.1186/s13024-020-00372-w

**Published:** 2020-04-15

**Authors:** Hui Chen, Yang Deng, Xiaoliang Gan, Yonghao Li, Wenyong Huang, Lin Lu, Lai Wei, Lishi Su, Jiawen Luo, Bin Zou, Yanhua Hong, Yihai Cao, Yizhi Liu, Wei Chi

**Affiliations:** 1 State Key Laboratory of Ophthalmology, Zhongshan Ophthalmic Center, Sun Yat-sen University, Guangzhou, 510060 China; 2 Department of Microbiology, Tumor and Cell Biology, Karoslinska Institute, 17177 Stockholm, Sweden

**Keywords:** Acute glaucoma, pyroptosis, NOD-like receptor 12, caspase-8/ HIF-1α

## Abstract

**Background:**

Acute glaucoma, characterized by a sudden elevation in intraocular pressure (IOP) and retinal ganglion cells (RGCs) death, is a major cause of irreversible blindness worldwide that lacks approved effective therapies, validated treatment targets and clear molecular mechanisms. We sought to explore the potential molecular mechanisms underlying the causal link between high IOP and glaucomatous RGCs death.

**Methods:**

A murine retinal ischemia/ reperfusion (RIR) model and an in vitro oxygen and glucose deprivation/reoxygenation (OGDR) model were used to investigate the pathogenic mechanisms of acute glaucoma.

**Results:**

Our findings reveal a novel mechanism of microglia-induced pyroptosis-mediated RGCs death associated with glaucomatous vision loss. Genetic deletion of gasdermin D (GSDMD), the effector of pyroptosis, markedly ameliorated the RGCs death and retinal tissue damage in acute glaucoma. Moreover, GSDMD cleavage of microglial cells was dependent on caspase-8 (CASP8)-hypoxia-inducible factor-1α (HIF-1α) signaling. Mechanistically, the newly identified nucleotide-binding leucine-rich repeat-containing receptor (NLR) family pyrin domain-containing 12 (NLRP12) collaborated with NLR family pyrin domain-containing 3 (NLRP3) and NLR family CARD domain-containing protein 4 (NLRC4) downstream of the CASP8-HIF-1α axis, to elicit pyroptotic processes and interleukin-1β (IL-1β) maturation through caspase-1 activation, facilitating pyroptosis and neuroinflammation in acute glaucoma. Interestingly, processing of IL-1β in turn magnified the CASP8-HIF-1α-NLRP12/NLRP3/NLRC4-pyroptosis circuit to accelerate inflammatory cascades.

**Conclusions:**

These data not only indicate that the collaborative effects of NLRP12, NLRP3 and NLRC4 on pyroptosis are responsible for RGCs death, but also shed novel mechanistic insights into microglial pyroptosis, paving novel therapeutic avenues for the treatment of glaucoma-induced irreversible vision loss through simultaneously targeting of pyroptosis.

## Background

Acute glaucoma, which remains the most common glaucoma type among Asians, is predicted to affect 20 million people worldwide and is characterized by a sudden and substantial increase in intraocular pressure (IOP), severe eye pain, and irreversible vision loss that can progress to permanent blindness [1–3]. The rapid elevation in IOP leads to retinal ischemia/ reperfusion (RIR) injury and retinal ganglion cells (RGCs) death [4]. The retina becomes vulnerable when disruption-induced ischemia/hypoxia in vessels results in shortages of oxygen and glucose, subsequently triggering a cascade of inflammatory processes [5]. Similar to the central nervous system (CNS), microglial cells are phagocytic sentinels in the retina required for neuronal homeostasis and innate immune defense, and their activation is responsible for neurodegenerative diseases [6]. Recently, we [7], Vargas et al. [8] and many other scientists [9–11] have reported that high IOP-induced ischemia initiates microglial neuroinflammation that mediates retinal tissue damage and RGCs death. It is demonstrated that retinal ischemia not only directly induces RGCs death but also triggers damage-associated molecular pattern (DAMP)-induced toll-like receptor 4 (TLR4) inflammasome-dependent neuroinflammation to activate the microglia, thus inducing further RGCs death [7, 12, 13].

Traditionally, cell death has been divided into several distinct subtypes such as apoptosis, necrosis and autophagy. Pyroptosis is a newly discovered form of programmed lytic cell death that is induced by inflammatory casapse-1 (CASP1), and is characterized by swelling of the cell, pore formation in unilamellar liposomes, membrane rupture and the release of inflammatory cytokines [14–18]. Gasdermin D (GSDMD) was discovered to be a substrate of CASP1, and cleavage of the N-terminal fragment of GSDMD forms membrane pores and drives pyroptosis [19–21]. Inflammasome complexes function as crucial intracellular effectors to initiate innate immunity and thus to defend against infections or tissue damage [22, 23]. It has been demonstrated that nucleotide-binding leucine-rich repeat containing receptor (NLR) family pyrin domain-containing 3 (NLRP3) and NLR family CARD domain-containing protein 4 (NLRC4) can both can form multiprotein complexes called inflammasomes, to activate CASP1, which subsequently cleaves the pore-forming protein GSDMD [15, 24, 25]. Previous studies have shown that the recently discovered NLR family pyrin domain-containing 12 (NLRP12) plays an important role in regulating the gut microbiota and autoinflammatory disease [26, 27]. However, the exact functions of NLRP12 in acute glaucoma are poorly studied and largely unknown. Furthermore, the precise functions and complex molecular mechanisms that occur between the novel molecule NLRP12 and pyroptosis remain elusive. Scientific research so far has focused on pathogen-induced pyroptosis [28, 29], prompting us to further investigate the involvement and mechanisms of damage-induced pyroptosis and the potential roles and mechanisms of NLRP12 in pyroptosis in an acute glaucoma model.

Hypoxia activates hypoxia-inducible factor-1α (HIF-1α) signaling, which activates a series of cellular processes including metabolic changes and angiogenesis to maintain cell viability [30–34]. HIF-1α drives interleukin (IL)-1β secretion to promote acute inflammatory responses in mouse primary cell cultures and C57BL/6 mice following systemic lipopolysaccharide (LPS) injection [35, 36]. Furthermore, we have also reported that caspase-8 (CASP8)-induced NLRP3 promotes IL-1β processing during innate immune responses in an IOP-induced retinal ischemia model [7]. Since both CASP8-induced NLRP3 and HIF-1α regulate IL-1β, we therefore explored the potential functions and underlying mechanisms of CASP8, NLRs, and HIF-1α with respect to the development of RIR injury, such as that in acute glaucoma.

In this study, we elucidated novel mechanisms for RGCs death and retinal tissue injury in acute glaucoma. We found that genetically deleting GSDMD substantially relieved RGCs death and the severity of retinal ischemic injury, suggesting the crucial functions of pyroptosis in the development of acute glaucoma. Our findings further revealed that NLRP12 cooperated with NLRP3 and NLRC4 to promote pyroptosis through CASP1-dependent GSDMD cleavage, lactate dehydrogenase (LDH) release and mature IL-1β processing. Notably, we found that NLRP12-, NLRP3- and NLRC4-induced CASP1 activation and pyroptosis were initiated by ischemia-triggered CASP8-HIF-1α signaling activation. Strikingly, bioactive IL-1β served as a key molecule that in turn positively accelerated the CASP8-HIF-1α-NLRP12/NLRP3/NLRC4 axis and promoted pyroptosis by cleaving GSDMD, indicating that IL-1β functioned as a pivotal node that magnified pyroptosis and neuroinflammation in the development of acute glaucoma.

## Materials and methods

### Antibodies and reagents

Antibodies targeting the following proteins were purchased from ABclonal Biotech Co., Ltd. (USA): HIF-1α (#A11945), HIF-1α (#A1544), NLRP3 (#A6345), NLRP12 (#A6671), NLRC4 (#A7382), CASP1 (#A0964), CASP8 (#A0215), IL-1β (#A1112) and GSDMD (#A10164). Anti-cleaved-CASP8 (#8592S) and anti-phospho-NF-kB P65 (#3033S) antibodies were obtained from Cell Signaling Technology. An IL-1β neutralizing antibody (#14–7012-85) was obtained from eBioscience. The CASP1 inhibitor (CASP1 inh) Z-YVAD-FMK (YVAD, #ab141388) was obtained from Abcam. The NF-kB P65 inhibitor JSH-23 (#S7351) was purchased from Selleckchem. Anti-β-actin (#AP0060) and secondary antibodies were purchased from Bioworld Technology. Lipofectamine 3000 was purchased from Invitrogen.

### Animal model of retinal ischemic injury

Adult female C57BL/6 mice and NLRP12^−/−^ mice and GSDMD^−/−^mice were purchased from the Model Animal Research Center at Nanjing University. The mice were anesthetized with 100 mg/kg pentobarbital sodium by intraperitoneal injection and then topically treated with 0.5% proparacaine (SomnoSuite; Kent Scientific, Torrington, CT, USA). The corneas were subsequently treated with 1% tropicamide (SomnoSuite; Kent Scientific) to dilate the pupils. Cannulation of the right eye in the anterior chamber was achieved by inserting a 30-gauge needle (BD, Franklin Lakes, USA) attached to a normal saline reservoir to elevate and maintain an average IOP of 110 mmHg for 90 min, which was monitored by our modified system according to Stockslager MA’s study [37]. The 30-gauge needle and attached normal saline reservoir were connected to a pressure transducer (Honeywell 142PC01G) attached to a three-way stopcock. Once the needle was inserted into the anterior chamber, we were able to record IOP in real time. We adjusted the height of the reservoir to maintain a stable IOP of 110 mmHg. The contralateral eye served as the sham group without an elevated IOP. After 90 min, the needle was withdrawn, and the IOP returned to normal levels. Tobramycin ointment was applied to the treated eyes to prevent infection. Eyes with cannulation-induced cataracts, iris injury/ bleeding or anterior chamber leakage were excluded from this study. No randomization was used.

### Genotyping of knockout (KO) mice

Mouse tail tissue was collected, and genomic DNA was isolated, mixed and incubated with a commercial kit DNeasy® Blood & Tissue Kit (Qiagen) according to the manufacturer’s instructions. A final centrifugation for 1 min at 6000×g was performed and the concentration of total DNA was measured by a NanoDrop ND-1000 Spectrophotometer (Thermo Scientific). The primers are listed as below.

NLRP12 primers:

Forward: TGGCTTCTATTCAACTCCCT.

Reverse: ATCGTTACACTCGGCTTCTC.

GSDMD primers:

Forward: CGATGGAACGTAGTGCTGTG.

Reverse: TCCTTCCCAACCTGCTGTTG.

The DNA sequences were verified by PCR and sequencing.

After PCR amplification, the samples were electrophoresed in 2% agarose 1 × TAE (Tris [40 mM Tris], acetic acid [20 mM] and ethylenediaminetetraacetic acid [1 mM]) gels, and stained with 1 × SYBR® Safe DNA Gel Stain (Life Technologies). Images were collected by an Image Lab system (Bio-Rad).

### Treatment regimen

After withdrawal of the needle from the anterior chamber, each experimental eye was injected with negative control (NC) small interfering RNA (siRNA)(20 μΜ/2 μL), CASP8 siRNA (20 μΜ/2 μL), NLRP3 siRNA (20 μΜ/2 μL), NLRC4 siRNA (20 μΜ/2 μL), GSDMD siRNA (20 μΜ/2 μL) or a CASP1 inh YVAD (200 μM/2 μL, Abcam, USA) into the vitreous cavity. The IOP returned to a normal level, and reperfusion was initiated. The mice were sacrificed 7 days after intravitreal siRNA injection.

### RNA sequence analysis

RNA was extracted from retinal tissue samples of C57BL/6 mice with and without RIR injury using a MasterPure Complete DNA and RNA Purification Kit (MC85200, Epicentre, Madison, WI, USA) according to the manufacturer’s instructions.

Briefly, 100 ng of RNA was sonicated into 300- to 400-bp fragments using a Bioruptor (Diagenode, Belgium), and sequencing libraries were prepared using a VAHTS Total RNA-seq (H/M/R) Library Prep Kit for Illumina® (NR603–02, Vazyme, Nanjing, China) following the manufacturer’s protocol. The RNA libraries were sequenced on an Illumina HiSeq2500 sequencer using a HiSeq SR Cluster Kit V4 (GD-401-4001, Illumina, San Diego, CA, USA) and a HiSeq SBS Kit V4 50 cycle kit (FC-401-4002, Illumina, San Diego, CA, USA). Initial processing was performed with CASAVA (v1.8.2, Illumina, USA).

After quality control with FastQC (v0.11.5), we used Cutadapt (v1.9.1) to trim low-quality bases. Trimmed reads were then aligned with TopHat (v2.0.11) to the mouse genome mm9, and unique mapped reads were obtained though filtering of the NH tags of bam files. We used Cufflinks (v2.1.1) to calculate gene expression values (as FPKM values) with the RefSeq mm9 reference transcriptome. Then, we summarized each sample’s expression values to detect significant differences in gene transcript expression between groups.

### Histological evaluation

After the mice were sacrificed, their eyes were enucleated at the designated time points, fixed with 4% paraformaldehyde (PFA) and paraffin embedded. Three sections across the optic nerve of each eye were prepared at 5-μm thickness and stained with hematoxylin and eosin staining (HE). The observational area was from the internal to the outer limiting membrane within a 1-mm distance from the optic nerve.

### Immunofluorescence staining of the retina

The mouse eyes were enucleated and embedded in optimal cutting temperature (OCT) compound, frozen and sectioned to a thickness of 6 μm. Anti-RBPMS (1:400) (#ab152101, Abcam, USA) was used to identify RGCs in the retina. DAPI was used for nuclear counterstaining. Images were collected by Olympus immunofluorescence microscope.

### Fluoro-gold (FG) labeling of RGCs

Mice were intraperitoneally anesthetized with a mixture of 100 mg/kg ketamine and 10 mg/kg xylazine. Ointment was applied to both eyes to prevent the corneas from drying. Next, to label RGCs, 4% fluoro-gold (FG) was injected into both sides of the superior colliculi 7 days prior to the establishment of RIR. The mice were sacrificed at the designated time points after reperfusion, and their eyes were fixed with 4% PFA on ice for 30 min before retinal flat mounting. FG-positive cells were subsequently identified by two independent observers using cellSens Dimension software (Olympus).

### Preparation of siRNAs

siRNAs targeting mouse NLRP3, NLRP12, NLRC4, HIF-1α, CASP8, GSDMD were designed by Ribobio Co., Ltd., using the mRNA sequences for these genes in the NCBI database as follows: mNLRP3 (GTACTTAAATCGTGAAACA), mNLRC4 (GCTGGGAGTTTGATGACTA), mNLRP12 (CCAAATGGAGACCCTCTTT), m HIF-1α (GGTATGTGGCATTTATTTG), mCASP8 (GTCATGCTCTATCAGATTT) and mGSDMD (CCATGGCCTCAATGTGCTT). The NC siRNA was purchased from Ribobio Co., Ltd. as well.

### Generation of CASP8 KO cell lines using the CRISPR/Cas9 system

Stable KO of CASP8 in BV2 microglial cells was established by BIOCYTOGEN. The sgRNAs targeting exon 3 to exon 5 of the wide-type (WT) allele were designed using an online tool to identify the guide sequences. The selected sequences were individually inserted into the CRISPR vectors, and the sequences for the sgRNAs used in the present study will be provided upon request. Cells were transfected with the CRISPR vectors and selected for 72 h in medium containing puromycin (0.75 g/mL). Subsequently single clones were selected through serial dilution. The clones were verified by PCR and western blot analysis.

### Cell culture and siRNA transfection

The BV2 microglial cell line was purchased from Procell Co., Ltd. (#CL-0493, Wuhan, China) and has been authenticated by Microread Genetics Co., Ltd. (Beijing, China) using PCR amplification. No mycoplasma contamination was found in this cell line. The cells were cultured in Dulbecco’s modified Eagle’s medium (DMEM) supplemented with 10% fetal bovine serum (FBS) and maintained at 37 °C in an incubator at 5% CO_2_. The cells were transfected using Lipofectamine 3000 (Invitrogen, USA) according to the manufacturer’s protocol. Microglial cells were cultured to 70% confluence in 6-well plates and transfected with NC siRNA and siRNAs (20 μM) targeting NLRP3, NLRP12, or NLRC4 and HIF-1α 12 h prior to oxygen and glucose deprivation/reoxygenation (OGDR) treatment. The cells were harvested 24 h after reperfusion, and cell lysates were obtained for protein assays.

### Culture of murine primary microglia

The cerebral cortices without meninges and blood vessels were separated from P0 – P2 newborn C57BL/6 J mice, followed by digestion with 0.125% trypsin for 15 min. Cells were isolated and plated on 25 cm^2^ flasks coated with poly – L – lysine (5 μg/mL).

Microglial cells were dissociated from the mixed cultures and collected on the 11th or 12th day by gently shaking and centrifugated. Purification was confirmed by the microglial marker IBA-1.

### OGDR treatment

To mimic the murine model of RIR, BV2 microglial cells were subjected to oxygen and glucose deprivation for 3 h and subsequently returned to their normal environment (37 °C, normoxic) and normal medium (DMEM supplemented with 10% FBS and glucose [4.9 g/L]) for 24 h prior to harvest. The cells were transferred into serum- and glucose-free medium and placed in a chamber (ProOx 110, Biospherix, USA) under hypoxic conditions (5% CO_2_ and 95% N_2_) in a 37 °C incubator for 3 h. Reperfusion was performed by exposing the cells to normoxic culture conditions and culturing them in DMEM supplemented with 10% FBS and glucose (4.9 g/L) in a 37 °C incubator for 24 h.

### Immunocytochemistry staining

WT and CASP8 KO BV2 microglial cells were subjected to oxygen and glucose deprivation for 3 h followed by 24 h of OGDR. After OGDR, the cells were fixed with 4% PFA for 30 min and washed several times with PBS. The primary antibodies targeted CASP8 (1:200), HIF-1α (1:50) and p-P65 (1:50). Cell nuclei were stained with DAPI. Fluorescence images were acquired by two independent observers with an Olympus fluorescence microscope.

### Western blot analysis

Total protein was isolated from either microglial cells or retinal tissue and processed for western blotting. Equal amounts of protein (50 μg for cell culture and 100 μg for retinal tissue) were loaded onto SDS-PAGE gels and transferred to polyvinylidene difluoride (PVDF) membranes. The membranes were blocked for 1 h with 5% nonfat dry milk diluted in TBST and subsequently incubated at 4 °C overnight with primary antibodies targeting the following proteins: HIF-1α (1:200), NLRP3 (1:500), NLRP12 (1:200), NLRC4 (1:500), CASP1 (1:200), CASP8 (1:200), IL-1β (1:200), GSDMD (1:500) and β-actin (1:1000). The following day, the blots were incubated with the appropriate secondary antibody (1:5000) for 1 h at room temperature, after which the blots were visualized with an enhanced chemiluminescence (ECL) kit, eBioscience) and recorded with an Image Lab imaging system (Bio-Rad, USA).

### Quantitative real-time PCR (RT-qPCR)

Total RNA was extracted from the WT BV2 and CASP8 KO BV2 microglial cells and mouse retinal tissues using TRIzol reagent (Invitrogen), and cDNA was synthesized with a PrimeScript RT Master Mix (TaKaRa). Quantitative amplification of murine HIF-1α, NLRP3, NLRP12, NLRC4 and β-actin was performed using a Light Cycler 480 Real-Time PCR System with software version LCS480 1.5.1.62. The mRNA levels of the target genes were normalized to those of β-actin. The primers of the target genes were as follows: mβ-actin (forward: GGCTGTATTCCCCTCCATCG; reverse: CCAGTTGGTAACAATGCCATGT), mHIF-1α (forward: GGCTCCCTTTTTCAAGCAGC; reverse: TGCTCCGTTCCATTCTGTTCA), mNLRP3 (forward: TTCTGCACCCGGACTGTAAA; reverse: TCGCCAAGATCATTGTTGCC), mNLRP12 (forward: GTTACACTCGGCTTCTCCT; reverse: GTTCTTCGTCTGGCTCAA) and mNLRC4 (forward: ACCTGGAAAAGGATGGGAATGAA; reverse: AAGTTTGGCAAGTCCTGGGG).

### Cytotoxicity assay, IL-1β ELISA and CASP8 activity assays

Cellular cytotoxicity was measured with an LDH release kit (#C0016, Beyotime, China) according to the manufacturer’s instructions. Cells were harvested at 24 h post-OGDR treatment, washed with cold PBS and centrifuged at low speed for 20 min. The supernatants were transferred to fresh tubes to measure the production of mature IL-1β using an IL-1β ELISA kit (Elabscience, USA). To assess CASP8 activity, the protein concentrations in BV2 cell lysates were measured with a BCA kit, and equal amounts of protein were mixed with the reaction reagent and added to 96-well plates for measurement with the CASP8 activity kit (BioVision, USA).

### Scanning electron microscopy (SEM)

WT and CASP8 KO BV2 microglial cells were fixed in 2.5% glutaraldehyde in phosphate buffer at 4 °C overnight. After the cells were dehydrated through critical point drying, they were coated with gold-palladium and examined with a scanning electron microscope (#FEIQuanta200; FEI, Co., Ltd., Czech Republic).

### Statistics

The data are shown as the mean ± SD. One-way ANOVA followed by Bonferroni multiple comparison tests was used to compare three or more groups, and two-tailed unpaired t-test was used to assess two groups using GraphPad Prism software (version 6.0, GraphPad Software, Inc., San Diego, CA, USA). *P* values less than 0.05 were considered to indicate statistical significance. The variance was similar among groups that were statistically compared.

## Results

### Pyroptosis plays a key role in elevated IOP-induced retinal ischemic injury and glaucomatous RGCs death

**Fig. 1.**
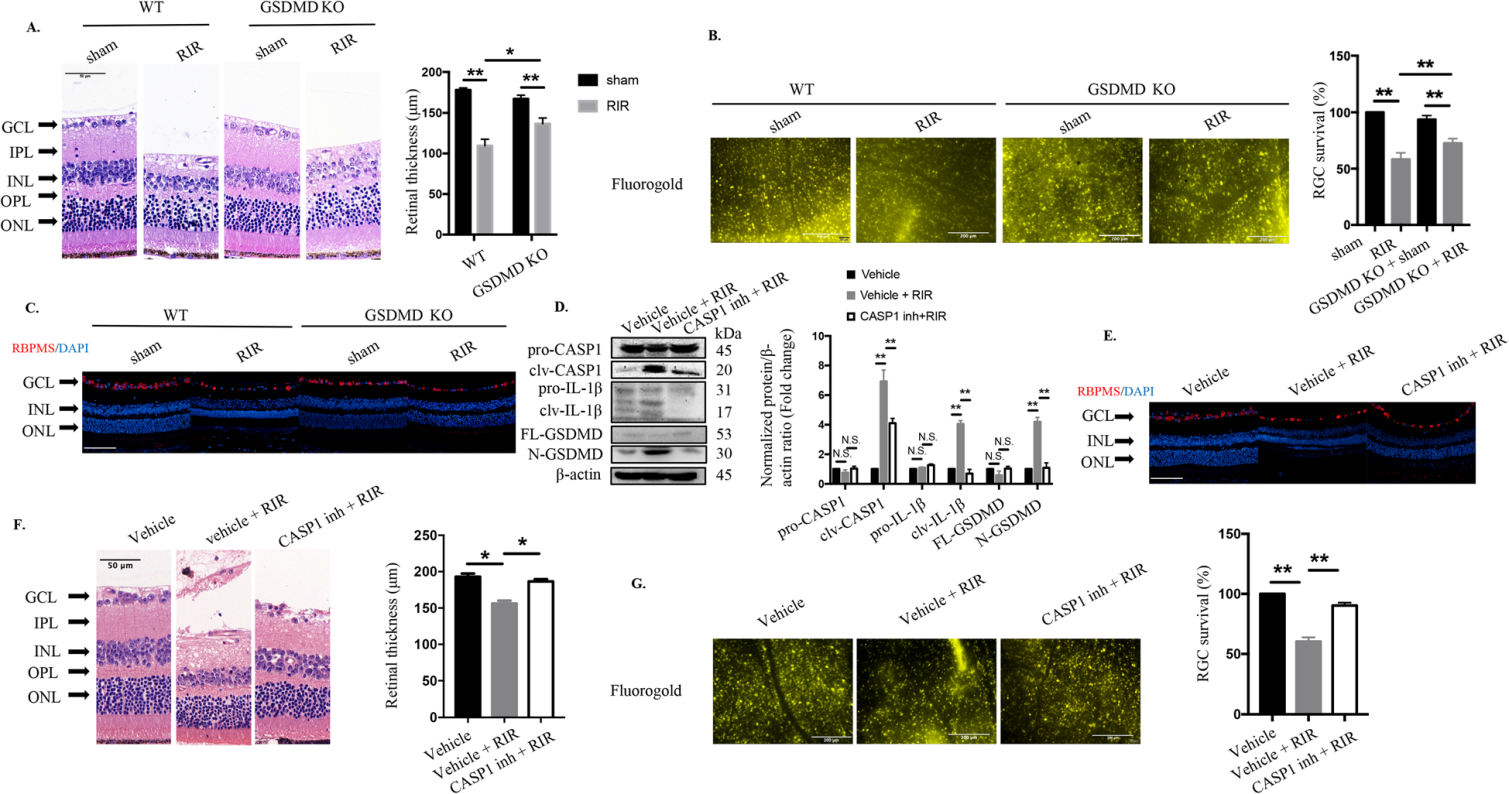
CASP1-dependent pyroptosis plays a vital role in elevated IOP-induced retinal ischemic damage and RGCs loss. **a** HE staining and quantitative analysis of total retinal thickness in retina tissue harvested 7 days post RIR injury (*n* = 6). Scale bar: 50 μm. **b** Retrograde FG-labeled images and quantitative assay of RGCs survival in WT and GSDMD KO mice (*n* = 6). Scale bar: 200 μm. **c** Representative immunofluorescence images of RGCs in the retina. Anti-RBPMS was used to label RGCs (*n* = 6). Scale bar: 100 μm. **d** Western blot analysis of the indicated proteins (*n* = 6). The protein levels were normalized to β-actin levels. **e** Representative immunofluorescence images of RGCs in the retina from mice treated with or without CASP1 inhibitor. Anti-RBPMS was used to label RGCs (*n* = 6). Scale bar: 100 μm. **f** HE staining and quantitative analysis of total retinal thickness in retinal tissue (*n* = 6). Scale bar: 50 μm. **g** Retrograde FG-stained images and quantitative assay of RGCs amount from groups subjected to RIR and RIR concomitant with CASP1 suppression groups (200 μΜ, *n* = 6). Scale bar: 200 μm. WT: wide type; KO: knockout; RIR: retinal ischemia-reperfusion; GCL: ganglion cell layer; IPL: inner plexiform layer; INL: inner nuclear layer; OPL: outer plexiform layer; ONL: outer nuclear layer; CASP1 inh: caspase-1 inhibitor. All the experiments are representative of at least three independent experiments. Data are represented as the mean ± SD. *P < 0.05, **P < 0.01, one-way ANOVA and two-way ANOVA

Pyroptosis is a novel inflammatory form of cell death triggered by CASP1/CASP11 and is characterized by membrane pore formation via the N-terminal fragment of GSDMD along with inflammatory cytokine leakage [19, 38]. Nevertheless, whether and how pyroptosis plays its roles in acute glaucoma are still unclear. We therefore investigated the exact functions of pyroptosis using genetic deletion approaches or pharmacological inhibitosr targeting pyroptosis-associated components in elevated IOP-induced RIR. Loss-of-function experiments showed that genetic elimination of the pyroptosis-effector GSDMD markedly increased total retinal thickness (136.7 ± 5 μm vs 109.6 ± 5.65 μm) and attenuated RGCs death after being subjected to RIR injury (Fig. 1a to c). As GSDMD was determined to participate in the pathogenesis of RIR, we therefore explored its upstream regulator. YVAD, the specific inhibitor targeting CASP1 (referred as CASP1 inh throughout the manuscript), significantly blocked the cleavage of IL-1β and GSDMD, indicating that RIR initiated pyroptosis via the canonical CASP1 pathway (Fig. 1 d). Furthermore, selective knockdown of CASP1 through intravitreal injection markedly attenuated the severity of retinal damage and restored RGCs numbers during RIR injury (Fig. 1e to g). These findings validated the pivotal roles of pyroptosis in aggravating retinal ischemic injury and RGCs death. Microglia are the most important type of cells in mediating immune responses in retinal tissue [6]. In this study, we established an in vitro model using BV2 microglia subjected to OGDR to mimic RIR injury. The occurrence of pyroptotic cell death in BV2 microglia subjected to ischemic injury was detected by SEM and was characterized by cell blebbing, cell swelling and cell flattening. Collectively, these findings reveal that CASP1-mediated pyroptosis participates in RIR pathogenesis and is associated with RGCs death and retinal ischemic damage in acute glaucoma.

**Fig. 2:**
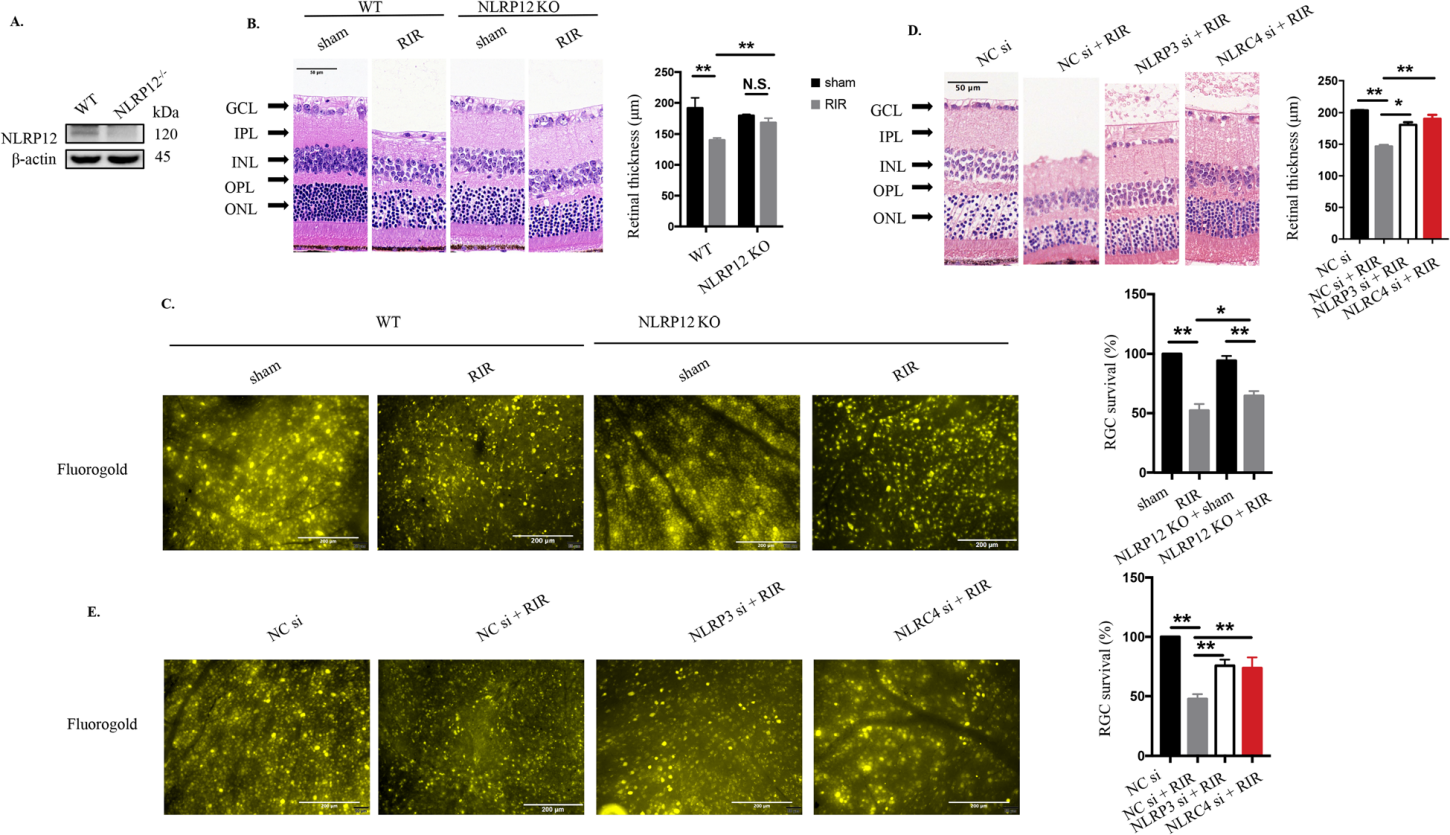
Genetic deletion of NLRP12 and inhibition of NLRP3 or NLRC4 significantly attenuate retinal damage and improve RGC survival. **a** Western blot detection of NLRP12 in retinas from NLRP12-deficient mice (n = 6). **b** HE staining and quantitative measurement of total retinal thickness targeting retinal tissue morphology in WT and NLRP12 KO mice under high IOP (n = 6). Scale bar: 50 μm. **c** Retrograde FG labeling and RGC amount evaluation in WT and NLRP12 KO mice under high IOP (n = 6). Scale bar: 200 μm. **d** HE staining and quantitative evaluation of total retinal thickness in retinal tissue subjected to high IOP followed by NLRP3/NLRC4 knockdown (20 μΜ, n = 6). Scale bar: 50 μm. **e** Retrograde FG labeling and quantitative measurement of RGC survival in retinal tissue subjected to elevated IOP followed by NLRP3/NLRC4 knockdown (20 μΜ, n = 6). Scale bar: 200 μm. WT: wide type; KO: knockout; RIR: retinal ischemia-reperfusion; GCL: ganglion cell layer; IPL: inner plexiform layer; INL: inner nuclear layer; OPL: outer plexiform layer; ONL: outer nuclear layer. All of the data are representative of at least three independent experiments. The data are represented as the mean ± SD. *P < 0.05, **P < 0.01, one-way ANOVA and two-way ANOVA.

### NLRP12, NLRP3, and NLRC4 are responsible for RIR injury-induced RGCs death

Our previous study has demonstrated that NLRP3 level is elevated in retinal tissue damage following retinal ischemia [7]. However, the exact roles of novel NLRs, NLRP12 and NLRC4 in acute glaucoma are still unresolved. In the current study, we found that high IOP-induced retinal ischemia promoted the upregulation of NLRP12 and NLRC4. We established an RIR model in NLRP12-deficient mice and found significant improvements in total retinal thickness and RGCs survival in NLRP12 KO mice compared with WT mice (Fig. 2 a to c and supplementary Fig. 1a). Furthermore, inhibition of NLRP3 or NLRC4 also markedly reduced the severity of retinal ischemic damage and improved RGCs survival (Fig. 2 d to e, and supplementary Fig. 1b). All these findings confirm that genetic deletion of NLRP12 or blockade of NLRP3 and NLRC4 protects retinal structure and RGCs from RIR-induced injury.

**Fig. 3.**
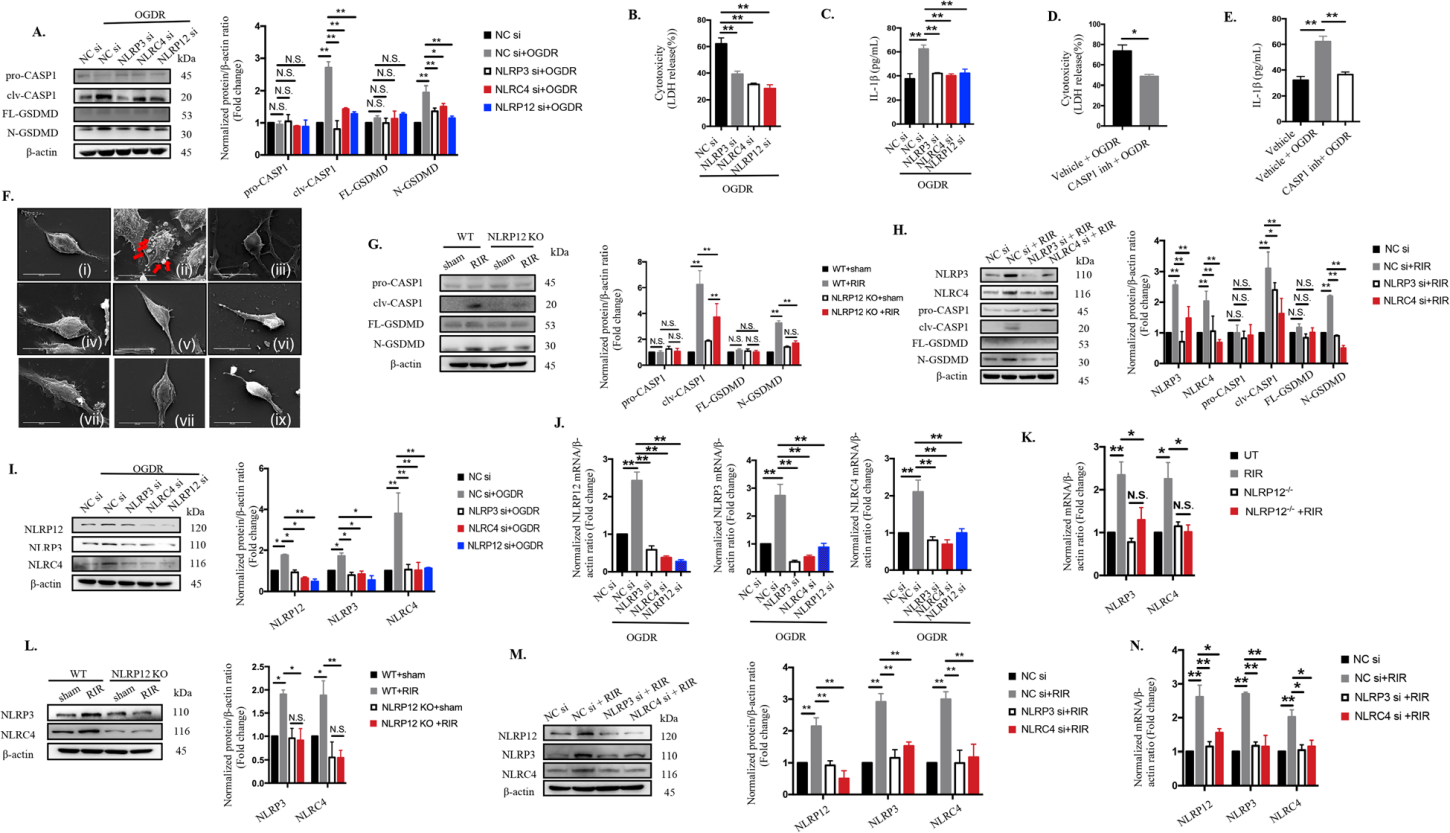
NLRP12 collaborates with NLRP3 and NLRC4 to elicit pyroptosis and promote IL-1β production in ischemic injury through CASP1-dependent GSDMD cleavage **a**-**h** NLRP12/NLRP3/NLRC4 promoted IL-1β release and induced pyroptosis by CASP1-dependent GSDMD cleavage: **a** Knockdown of NLRP12/NLRP3/NLRC4 reduced CASP1 and GSDMD cleavage in extracts from BV2 microglia after OGDR injury, as determined by western blot analysis (n = 6). The protein levels were normalized to β-actin levels. **b** LDH release (n = 6). **c** IL-1β secretion (n = 6). **d**-**e** LDH release (**d**) and IL-1β production (**e**) of BV2 microglia treated with the CASP1 inh, YVAD (200 µM) and subjected to OGDR injury (n = 6). **f **Representative SEM images showed pyroptotic cell death and other morphological changes in BV2 microglia subjected to OGDR combined with or without different additional treatments (n = 5): (i): control; (ii): OGDR; (iii): OGDR plus CASP1 inh (YVAD, 200 µM); (iv): OGDR plus NLRP3 siRNA (si); (v): OGDR plus NLRP12 si; (vi): OGDR plus NLRC4 si; (vii): CASP8 CRISPR plus OGDR; (viii): OGDR plus HIF-1α si ; (ix): OGDR plus IL-1β neutralizing antibody. Scale bar: 20 µm. **g**-**h** Immunoblotting analysis for detection of pyroptotic proteins in the retinas of NLRP12-/- or WT mice with or without NLRP3/NLRC4 knockdown under RIR conditions (n = 6). The protein levels were normalized to β-actin levels. **i**-**n** Mutual regulatory relationships among NLRP12, NLRP3 and NLRC4: **i**-**j** Protein expression and mRNA levels of the indicated molecules, as determined by western blotting and qRT-PCR detection in BV2 microglia exposed to OGDR with or without NLRP3/NLRP12/NLRC4 knockdown (n = 6). The mRNA and protein levels were normalized to β-actin levels. **k**-**n** Western blotting and qRT-PCR analyses of NLRP3, NLRP12, and NLRC4 in retinas from WT mice and NLRP12 KO mice that were sacrificed 7 days after elevated IOP injury, combined with or without NLRP3/NLRC4 knockdown (n = 6). The mRNA and protein levels were normalized to β-actin levels. The data shown are representative of at least three independent experiments. The data are represented as the mean ± SD. *P < 0.05, **P < 0.01, one-way ANOVA, two-way ANOVA and two-tailed unpaired t-test.

### NLRP12 collaborates with NLRP3 and NLRC4 to induce pyroptosis by CASP1-dependent GSDMD cleavage

The activation of NLRP3 recruits pro-CASP1 into inflammasomes and cleaves it into its active form, which subsequently induces GSDMD cleavage to drive pyroptotic cell death as a pathogen defense mechanism [39, 40]. However, the effects of other NLR inflammasomes on pyroptosis in response to danger-associated signals have not been reported. Consequently, we investigated the effects and regulatory mechanisms of NLRs on pyroptosis in vitro and in vivo. First of all, we established OGDR model using murine primary microglial cells (Supplementary Fig. 2a) and found that the levels of NLRP3/ NLRP12/ NLRC4 and the effector of pyroptosis, termed GSDMD, were significantly increased in OGDR group than that in the controls (Supplementary Fig. 2b-e). In an In vitro model using BV2 microglia subjected to OGDR, knocking down NLRP3, NLRP12, or NLRC4 suppressed neuroinflammation and pyroptosis through such effects as the downregulation of cleaved-CASP1, reductions in cleaved-GSDMD and mature IL-1β levels, and reductions in LDH leakage (Fig. 3 a to c). Similarly, NLRP12 KO and knockdown of NLRP3/NLRC4 reduced N-GSDMD and cleaved-CASP1 levels in the mouse model (Fig. 3g and h). Furthermore, inhibiting CASP1 significantly reduced pyroptosis-associated LDH release (Fig. 3d) and IL-1β secretion (Fig. 3e) in vitro, as well as N-GSDMD cleavage and IL-1β production (Fig. 1e) in vivo, suggesting that multiple NLRs drove pyroptotic death via CASP1 cleavage.

Here, we discovered the first evidence of reciprocal regulation among NLRP3, NLRP12, and NLRC4. Suppression of NLRP12 decreased the production of NLRP3 and NLRC4, while the knockdown of either NLRP3 or NLRC4 also reduced the production of the other two NLRs (Fig. 3i and j), indicating that NLRP12 acted synergistically with NLRP3 and NLRC4. The elevated IOP-induced retinal ischemic murine model also confirmed the in vitro data (Fig. 3 k to n).

Taken together, these results demonstrated that NLRP12 collaborates with NLRP3 and NLRC4 to induce pyroptosis through CASP1-dependent GSDMD cleavage during ischemic injury.

### CASP8 is involved in acute glaucoma

**Fig. 4:**
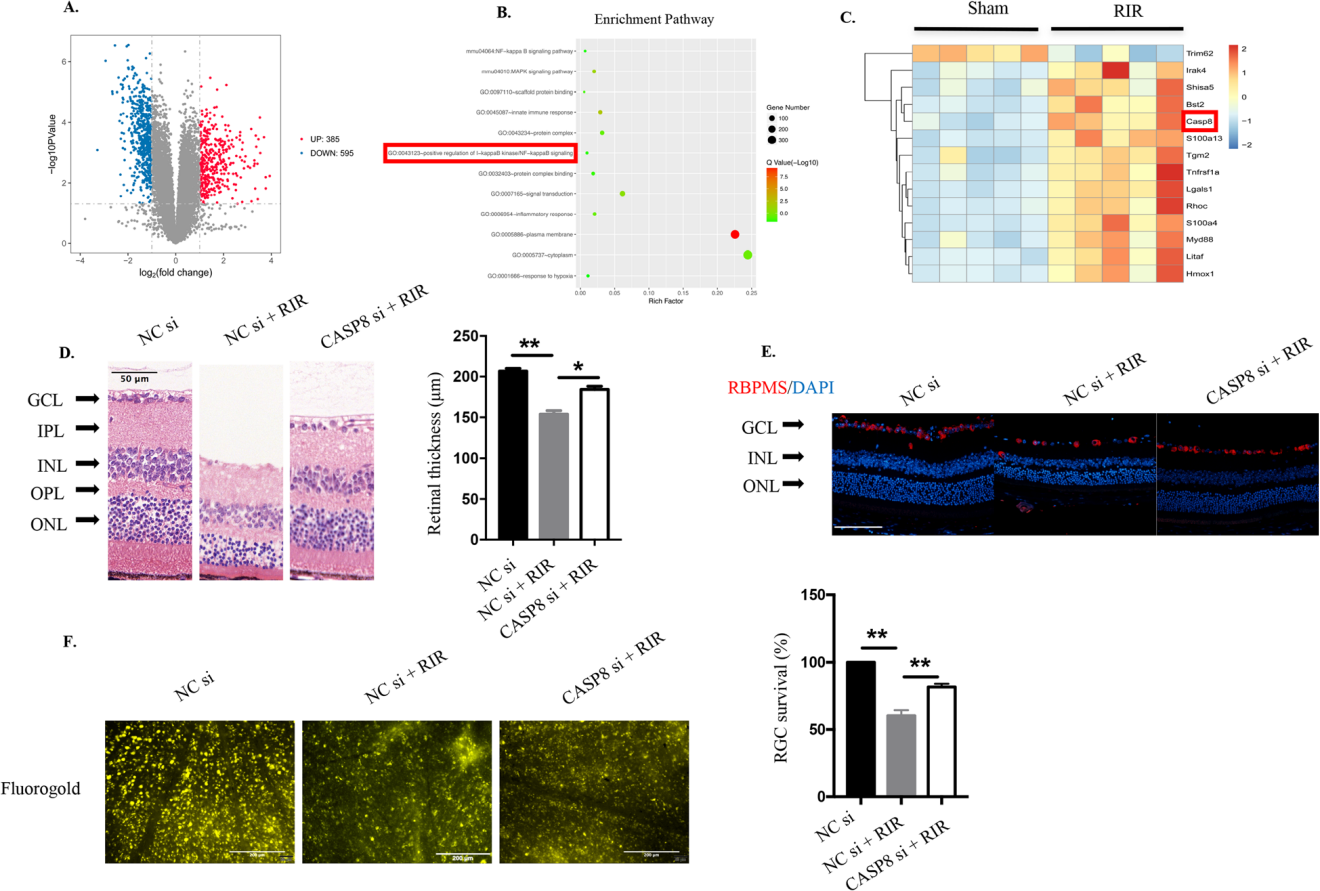
CASP8 is a pivotal participant in RIR injury. **a** Volcano plot showing a total of 980 genes, of which 385 were upregulated and 595 were downregulated in retinal tissue in the RIR group compared with the sham group. **A** fold change > 2 or < 0.5 was considered statistically significant (*n* = 5 mice/group). **b** KEGG pathway enrichment analysis of differentially expressed genes between the ischemia/reperfusion and sham groups (*n* = 5 mice/group). The enriched pathways of hypoxia and inflammatory signaling are shown. The positive regulation of I-kappaB kinase/NF-kappaB signaling was chosen for further analysis. **c** Heat map showing the contents of the positive regulation of I-kappaB kinase/NF-kappaB signaling in the retinas of mice after the RIR process (*n* = 5 mice/group). RIR mice were sacrificed 7 days after reperfusion, and retinal tissue was collected for RNA sequence analysis. **d** HE staining of retinas from mice under RIR with or without CASP8 knockdown (20 μΜ). The retinal tissue was harvested on the seventh day after reperfusion (*n* = 6). Scale bar: 50 μm. The total retinal thicknesses were quantified for the three groups (*n* = 6). **e** Representative immunofluorescence images of RGCs in the retina from mice treated with or without CASP8 knockdown. Primary antibody against RBPMS was used to label RGCs (*n* = 6). Scale bar: 100 μm. **f** Retrograde FG staining of RGCs from RIR injury in the absence or presence of CASP8 interference (20 μΜ, *n* = 6). Scale bar: 200 μm. RGCs survival were analyzed comparing to the controls (*n* = 6). RIR: retinal ischemia-reperfusion; GCL: ganglion cell layer; IPL: inner plexiform layer; INL: inner nuclear layer; OPL: outer plexiform layer; ONL: outer nuclear layer; CASP8 si: caspase-8 siRNA. All of the data are representative of at least three independent experiments. Data are represented as the mean ± SD. *P < 0.05, **P < 0.01, one-way ANOVA

To gain better insight into the pathological molecular changes initiated by high IOP-induced RIR injury, we performed RNA sequence analysis and Kyoto Encyclopedia of Genes and Genomes (KEGG) pathway enrichment analysis on retinal tissue from untreated and RIR mice. Notably, 385 genes were upregulated and 595 genes were downregulated in the RIR group as compared with the sham group (Fig. 4a). KEGG analyses showed that multiple pathways in response to inflammatory and hypoxia as well as signaling transduction pathways such as the NF-kappa B and MAPK signaling pathways were clearly activated during reperfusion (Fig. 4b). As the NF-kB pathway has been reported to positively involved in neuroinflammation [41, 42], we investigated the positive regulation of I-kappaB kinase/NF-kappaB signaling in this murine ischemia-reperfusion model. Among the factors involved in this indicated pathway, CASP8 was markedly upregulated at the mRNA level in the retinas of mice subjected to RIR injury (Fig. 4c), suggesting that CASP8 actively participates in the RIR pathogenesis.

CASP8 is considered crucial for embryonic development, and CASP8^−/−^ mice are found abnormal or close to death at 10.5 to 12.5 at embryonic days (E10.5 to E12.5) [43, 44]. We explored the underlying functions of CASP8 by intravitreally injecting CASP8 siRNA into elevated IOP-induced RIR model animals. Our findings revealed that knocking down CASP8 significantly attenuated retinal damage and reduced RGCs loss (Fig. 4d to f), indicating the pivotal role of CASP8 in mediating retinal ischemic injury.

### Inhibition of CASP8-mediated HIF-1α signaling protects the retina and RGCs from ischemic injury

**Fig. 5:**
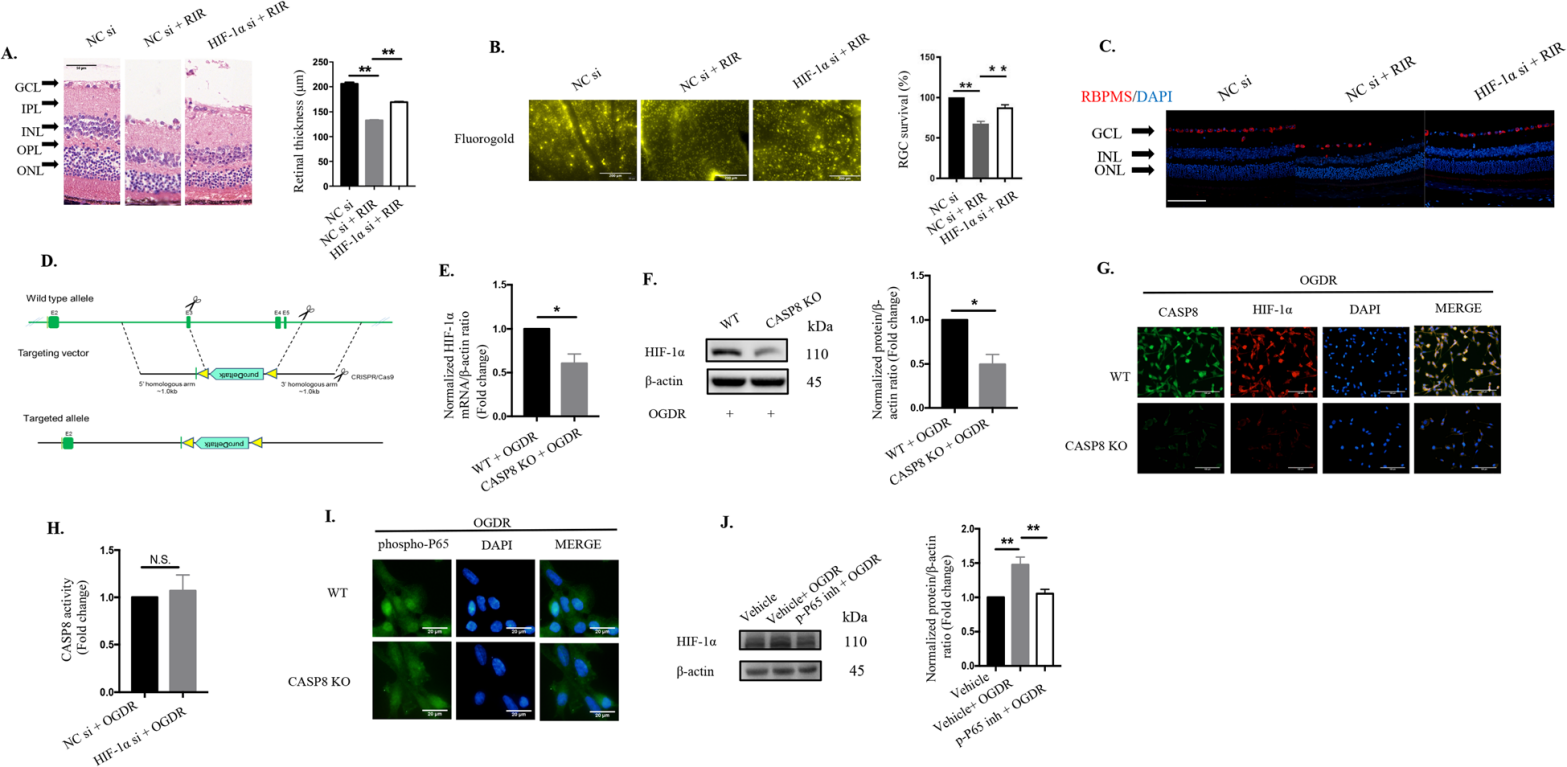
CASP8-mediated HIF-1α signaling is involved in retinal ischemic injury and RGCs loss. **a** HE staining and quantitative evaluation of total retinal thickness in retinal tissue subjected to high IOP followed by HIF-1α knockdown (20 μΜ, n = 6). Scale bar: 50 μm. **b** Retrograde FG labeling and quantitative measurement of RGCs survival from mice subjected to RIR injury in the absence or presence of HIF-1α interference (20 μΜ, n = 6). Scale bar: 200 μm. **c** Representative immunofluorescence images of RGCs in the retina from mice treated with or without HIF-1α blockage. Primary antibody against RBPMS was used to label RGCs (*n* = 6). Scale bar: 100 μm. **d** CRISPR-CAS9 design to knock out CASP8 in BV2 cell line. Targeted vector was designed based on the exon 3 to exon 5 in WT allele. **e-f** RNA level and protein levels of HIF-1α in WT and CASP8 KO BV2 cell line exposed to OGDR insult (*n* = 6, both). The protein and mRNA levels were normalized to β-actin levels. **g** BV2 microglia with the indicated genotypes were subjected to OGDR and stained with antibodies against cleaved-CASP8 and HIF-1α (*n* = 6). Scale bar: 100 μm. **h** CASP8 activity (*n* = 5). **i** Representative images of immunofluorescence staining targeting phospho-NF-kB P65 translocation in WT BV2 microglia and CASP8-specific KO cell line exposed to OGDR (*n* = 6). Scale bar: 20 μm. **j** The protein levels of HIF-1α were assayed by immunoblots in BV2 microglia treated with the NF-kB P65 inhibitor, JSH-23 (40 μM, *n* = 6). The protein expression was normalized to β-actin expression. RIR: retinal ischemia-reperfusion; GCL: ganglion cell layer; IPL: inner plexiform layer; INL: inner nuclear layer; OPL: outer plexiform layer; ONL: outer nuclear layer; HIF-1α si: HIF-1α siRNA. All of the data are representative of at least three independent experiments. Data are represented as the mean ± SD. *P < 0.05, **P < 0.01, one-way ANOVA and two-tailed unpaired t-test.

Research has increasingly shown that HIF-1α plays a critical role in regulating gene expression during the pathogenesis of retinal hypoxia/ischemia injury [45–48], which has been further confirmed in our study. Intravitreally knockdown of HIF-1α significantly alleviated retinal damage and RGCs loss (Fig. 5 a to c). These findings prompted us to explore whether there is a regulatory relationship between CASP8 and HIF-1α. Therefore, we established a CASP8 KO BV2 cell line using the CRISPR-CAS9 system (Fig. 5d) to determine the role of CASP8 under OGDR conditions. Our data showed that CASP8 KO significantly blocked HIF-1α expression under OGDR (Fig. 5e to g), whereas blockade of HIF-1α had no effect on CASP8 activity (Fig. 5h), indicating that CASP8 was upstream of HIF-1α signaling. We next explored the molecular regulatory mechanisms by which CASP8 controlled HIF-1α. NF-kB signaling plays a critical role in the pathogenesis of inflammation and our results showed that deletion of CASP8 effectively blocked NF-kB translocation (Fig. 5i), while inhibition of NF-κB transcriptional activity significantly downregulated HIF-1α expression (Fig. 5j). Taken together, these findings revealed that HIF-1α signaling is induced by CASP8 in this OGDR model via NF-kB translocation.

### NLRP12/NLRP3/NLRC4 and CASP1 activation is triggered by the CASP8-HIF-1α pathway

**Fig. 6:**
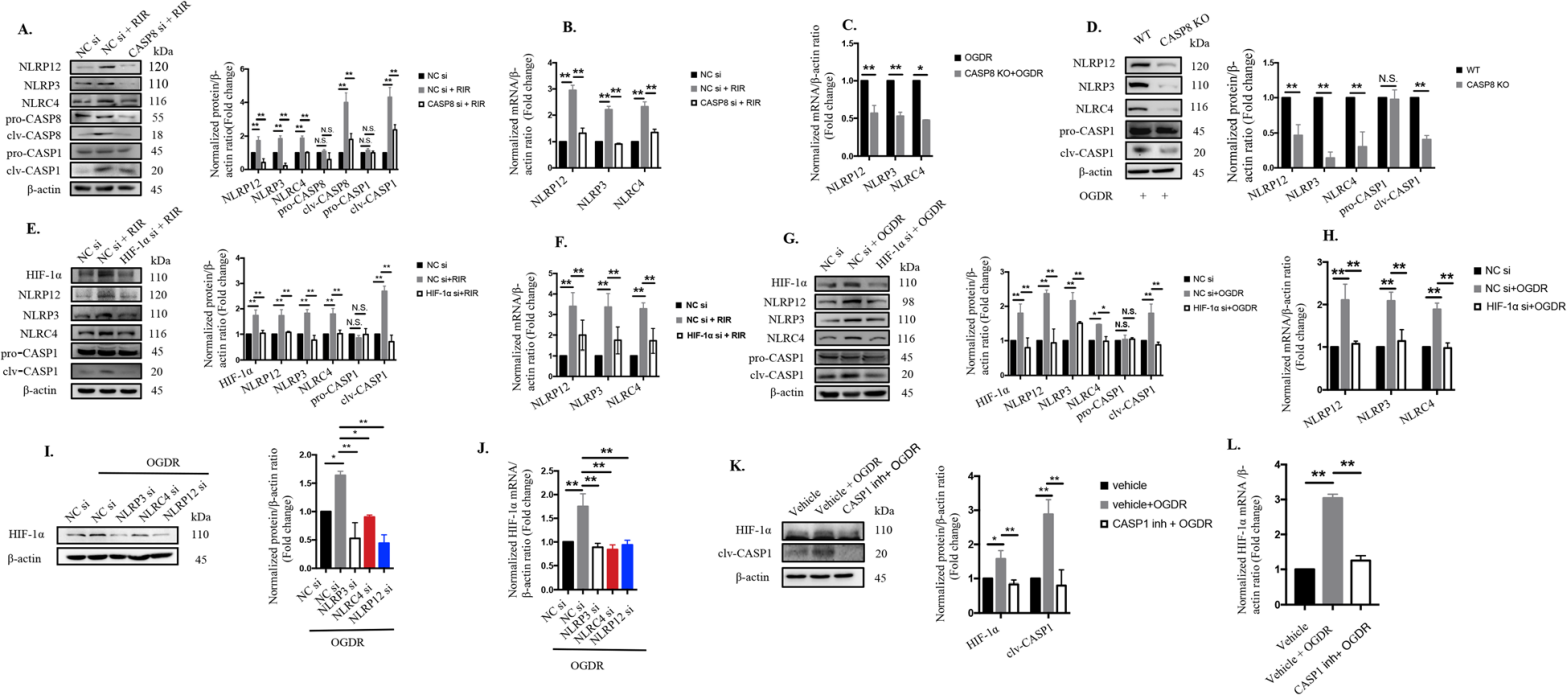
CASP8 promotes NLRP12/NLRP3/NLRC4 and CASP1 activation upon HIF-1α signaling. **a-b** The protein and mRNA levels of NLRP12/NLRP3/NLRC4 and CASP1 was detected in retinas from WT mice with or without CASP8 knockdown (20 μΜ) that were harvested at the seventh day after reperfusion (*n* = 6). The protein and mRNA levels were normalized to β-actin levels. **c-d** CASP8 elimination diminished the activation of NLRP12/NLRP3/NLRC4 and CASP1 in BV2 microglia exposed to OGDR (*n* = 6). The protein and mRNA levels were normalized to β-actin levels. **e-h** Immunoblot and qRT-PCR analyses of targeting NLRP12/NLRP3/NLRC4 and CASP1 in vivo and in vitro (n = 6) with or without HIF-1α knockdown. The protein and mRNA levels were normalized to β-actin levels. **i-j** Protein and mRNA levels of HIF-1α upon NLRP12/NLRP3/NLRC4 suppression in vitro (*n* = 6). The mRNA and protein levels were normalized to β-actin levels. **k-l** Knockdown of CASP1 suppressed the production of HIF-1α protein and mRNA in vitro (*n* = 6). The data shown are representative of at least three independent experiments. The data are represented as the mean ± SD. *P < 0.05, **P < 0.01, one-way ANOVA

We previously validated that CASP8 mediates retinal neuroinflammation by promoting the activation of NLRP1 and NLRP3 [7], whereas the effects of CASP8 on the newly discovered NLRs remain unknown. Therefore, we explored the influence of CASP8 on the newly-discovered NLRC4 and NLRP12 during RIR (Fig. 6a and b). The production of NLRP3, NLRC4, and NLRP12 was all significantly lower in WT mice treated with CASP8 siRNA than in control mice, indicating that CASP8 exerted its inflammatory effect on retinal innate immunity by activating not only the classic NLRP3 but also the novel NLRP12 and NLRC4 in retinal ischemia (Fig. 6a and b). Knocking out CASP8 also likewise suppressed NLRP12/NLRP3/NLRC4 upregulation and CASP1 cleavage in microglia subjected to OGDR (Fig. 6c and d), indicating that CASP8 activation was at least partially responsible for the activation of NLRP3/NLRP12/NLRC4 and CASP1.

Although CASP8 regulated HIF-1α signaling and NLRP3/NLRP12/NLRC4 activation, the details of the mutual regulation between HIF-1α and NLRs remains obscure. Accordingly, we investigated the precise relationships between HIF-1α and the NLRP3/NLRP12/NLRC4 inflammasomes. Our findings revealed that selective knockdown of HIF-1α was associated with reduced activation of NLRP3/NLRP12/NLRC4 and CASP1 activation in vivo and in vitro (Fig. 6e to h). In addition, knockdown of NLRP12, NLRP3 or NLRC4 or suppression of CASP1 in turn reduced the expression of HIF-1α (Fig. 6 i to l), suggesting that there was a positive feedback loop among NLRs, CASP1 and HIF-1α. Altogether, these results indicated that NLRP12/NLRP3/NLRC4 and CASP1 activation is induced by CASP8-modulated HIF-1α signaling.

### Activation of the CASP8-HIF-1α pathway elicits pyroptosis via CASP1-dependent GSDMD cleavage

**Fig. 7:**
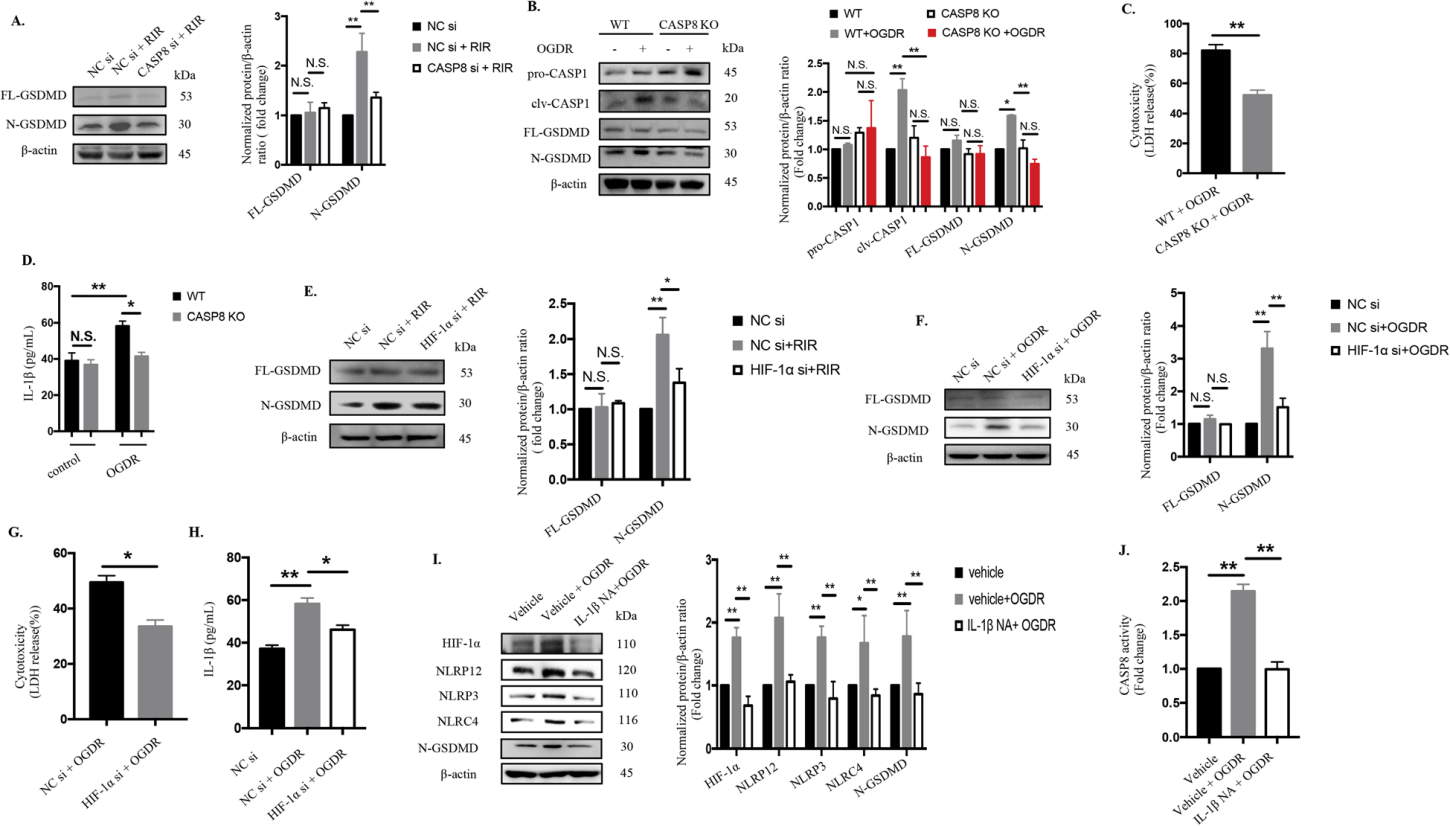
Activation of the CASP8-HIF-1α pathway elicits pyroptosis and promotes IL-1β production which in turn magnifies inflammatory cascades via the CASP8-HIF-1α-NLR axis. **a** The protein levels of cleaved GSDMD were detected in retinas from WT mice with or without CASP8 knockdown (20 μΜ) that were harvested at the seventh day after reperfusion (*n* = 6). The protein levels were normalized to β-actin levels. **b** Western blot analysis of cleaved CASP1 and GSDMD in extracts from WT BV2 microglia and CASP8-specific KO cell line after OGDR injury (*n* = 6). The protein levels were normalized to β-actin levels. **c-d** Cytotoxicity **c** and IL-1β production **d** in BV2 microglia under OGDR injury (*n* = 6). **e-f** The protein levels of cleaved GSDMD were detected in vivo and in vitro with or without HIF-1α knockdown. The protein levels were normalized to β-actin levels. **g-h** Cytotoxicity **g** and IL-1β processing **h** were measured in the presence or absence of HIF-1α siRNA treatment in BV2 microglia (*n* = 6, both). **i** Western blotting detection of the indicated proteins in BV2 microglia subjected to OGDR and OGDR concomitant with IL-1β neutralizing antibody treatment (*n* = 6). The protein levels were normalized to β-actin levels. **j** CASP8 activity (*n* = 6). The data shown are representative of at least three independent experiments. Data are represented as the mean ± SD. *P < 0.05, **P < 0.01, experiments were assessed by one-way ANOVA, two-way ANOVA or two-tailed unpaired t-test

Although CASP8 has traditionally been considered as a pivotal apoptotic initiator [49], accumulating studies have revealed the nonapoptotic roles of CASP8 as initiating cleavage of GSDMD during Yersinia infection [50, 51]. Nevertheless, the potential link between CASP8 and pyroptosis in DAMP-induced RIR injury is still unclear. Our current study verified that CASP8 inhibition led to a marked reduction in the cleavage of GSDMD (Fig. 7a), implying that CASP8 induced GSDMD-dependent pyroptosis during RIR pathogenesis.

In vitro, deletion of CASP8 also markedly suppressed CASP1 and GSDMD cleavage, cellular cytotoxicity, IL-1β release and pyroptotic cell death (Fig. 7 b, c, d and Fig. 3f). These data indicated that CASP8 not only participated in neuroinflammation via IL-1β secretion, but also mediated CASP1-induced pyroptosis under OGDR. Meanwhile, we are the first to report HIF-1α blockage reduced cleavage of GSDMD and IL-1β production as well as attenuated LDH leakage and pyroptotic cell death in vivo and in vitro (Fig. 7 e, f, g, h and Fig. 3f). Taken together, these results confirmed that the CASP8-HIF-1α pathway is critical to IL-1β processing and initiation of pyroptosis via NLR-activated CASP1 signaling in response to ischemic injury.

### Mature IL-1β is the key factor activating NLRP12/NLRP3/NLRC4-induced pyroptosis through CASP8-HIF-1α pathway

**Fig. 8:**
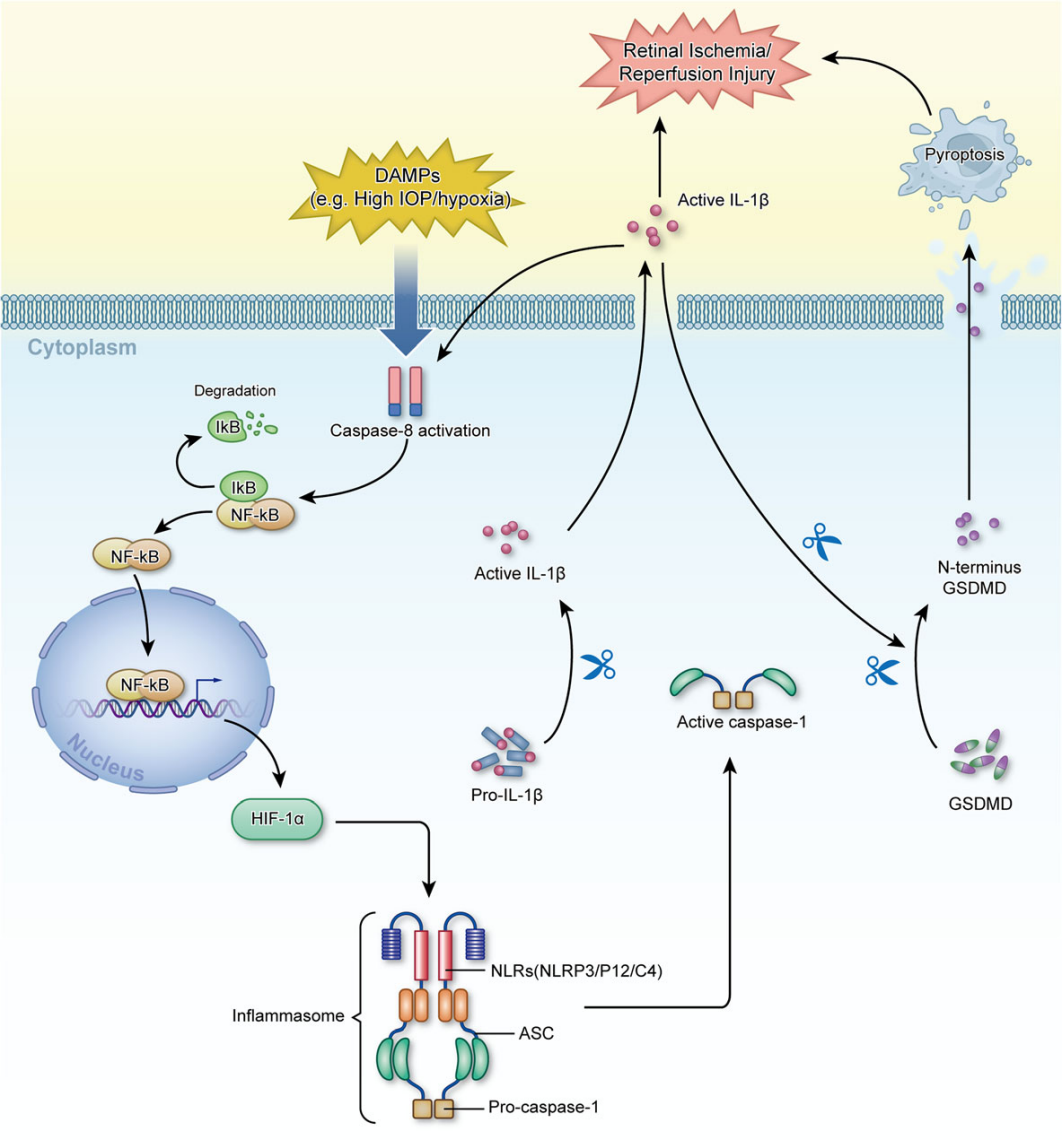
Diagram illustrating the pathway by which the CASP8-HIF-1α- NLRP12/NLRP3/NLRC4-IL1β-pyroptosis circuit contributes to the pathogenesis of acute glaucoma

HIF-1α production, NLRs expression and CASP8 activity were dramatically reduced when dissociative IL-1β was neutralized (Fig. 7i and j), indicating that IL-1β could in turn regulated the CASP8-HIF-1α-NLRs axis. It implied that CASP8-HIF-1α-NLRs axis-induced IL-1β maturation is a positive feedback loop to trigger inflammatory cascades. Furthermore, neutralizing IL-1β reduced the release of N-terminal GSDMD to protect against pyroptotic death (Fig. 7i and Fig. 3f). Overall, these results reveal a novel pathway whereby the CASP8-HIF-1α-NLRs-IL-1β axis mediates pyroptosis and neuroinflammation during the pathogenesis of retinal ischemic injury (Fig. 8). Additionally, these data further extend the functions of IL-1β in regulating CASP8, HIF-1α and NLRP3/NLRP12/NLRC4 axis and amplifying pyroptosis.

## Discussion

Acute glaucoma is one of the most common causes of sharp vision loss worldwide [2]. Elevated IOP induces retinal ischemia and RGCs loss, leading to irreversible visual impairment [52]. Medication aimed at lowering IOP to an appropriate level is regarded as the principle strategy for the treatment of acute glaucoma [53]. Unfortunately, there is a lack of approved, effective and validated treatment targets for acute glaucoma. Therefore, prevention and attenuation of acute glaucoma damage are unmet clinical needs. Previously, neuroinflammation in response to ischemia has been shown to play a pivotal role in the pathogenesis of elevated IOP-induced retinal ischemic injury and RGCs death [7, 8, 54]. A deeper understanding of the potential neuroinflammation mechanisms is urgently needed to support identification of key targets for acute glaucoma treatment.

In the current study, we demonstrated that pyroptosis plays crucial roles in promoting RGCs death and retinal ischemic injury in acute glaucoma. Remarkably, our findings reveal novel functions of NLRP12, which is induced by CASP8 and HIF-1α activation. Furthermore, we found that NLRP12 collaborates with NLRP3 and NLRC4 to initiate pyroptosis via CASP1-dependent GSDMD cleavage in vivo and in vitro. We also discovered a crucial regulatory association between CASP8 and HIF-1α in which CASP8 modulates HIF-1α signaling by driving NF-kB translocation. Furthermore, we found that IL-1β magnifies pyroptosis via promoting CASP8-HIF-1α-induced NLRP12/NLRP3/NLRC4 activation, indicating its role in a key positive feedback loop that accelerates neuroinflammation and pyroptosis.

Pyroptosis is a newly discovered cell death mechanism mediated by inflammasome-dependent CASP1 activation and is characterized by the presence of multiple pores in lipid membranes formed by the N-terminus of GSDMD [19, 20]. The precise function of GSDMD has attracted increasing attention. We therefore elucidated the role of GSDMD in DAMP-induced retinal injury using GSDMD-deficient mice subjected to substantial elevated IOP. Interestingly, our study firstly revealed, for the first time, that GSDMD elimination significantly improves RGCs survival and reduces the severity of retinal tissue injury, indicating that GSDMD-predominant pyroptosis may be a potential therapeutic target for RGCs loss and retinal tissue injury. Microglia are gatekeepers of neurons in the CNS, and overactivated microglia harm RGCs by releasing inflammatory cytokines and recruiting inflammatory effector cells to the insult area to magnify cascade of inflammation [55]. By means of an in vitro model mimicking the physiology of RIR injury, our study shows that microglial activation initiates pyroptosis by eliciting GSDMD cleavage, thus promoting IL-1β secretion and LDH release. These findings indicate that activated microglia-triggered GSDMD-dependent pyroptosis contributes to ischemia-induced RGCs death. One reasonable explanation for this finding is that disruption of GSDMD in microglia reduces pore formation in the membrane and reduces leakage of cytoplasmic components including bioactive IL-1β, thus ameliorating neuroinflammation and RGCs loss.

To gain better insight into the upstream molecular mechanisms of pyroptosis, we next focused on inflammasomes. NLRP3 is a classic sensor and has been reported to drive GSDMD cleavage in response to pathogen infection [56]; therefore, we investigated the roles of the newly discovered NLRs NLRP12 and NLRC4. Unlike that of NLRP3, the effect of NLRP12 has been controversial. Several reports state that NLRP12 functions as a negative regulator of inflammation in tumorigenesis [57–59], colitis [60, 61] and obesity [62], while Wang et al.report that NLRP12 can activate inflammatory signaling pathways by regulating the activation of NF-kB and CASP1-dependent cytokine processing [63]. In current study, we found for the first time that genetic deletion of NLRP12 strikingly attenuates the severity of RGCs loss and retinal damage, validating NLRP12 activation is a trigger for retinal ischemic injury in acute glaucoma. Moreover, NLRP12 was detected to collaborate with NLRP3 and NLRC4 to promote pyroptosis by eliciting CASP1-dependent N-terminal GSDMD cleavage. Together, these findings depict a novel function for NLRP12 in pyroptosis with regard to the pathogenesis of retinal ischemia and RGCs death, indicating that NLRP12 coordinates with NLRP3 and NLRC4 to exert both neuroinflammatory and pyroptotic effects under ischemic conditions.

Accumulating evidence shows that GSDMD prevents CASP8 activation at the NLRC4 or NLRP3 inflammasomes under conditions of exposure to a bacterial inflammasome activator [64, 65]. We thus investigated the possible link between CASP8 and GSDMD. Our findings reveal the unique effect of CASP8 on pyroptosis: CASP8 initiates GSDMD-predominant pyroptosis during retinal ischemia, in accordance with the results during pathogen infection [50, 51]. However, whether GSDMD in turn influences the activation of CASP8 in elevated IOP-induced retinal ischemic injury requires future study.

HIF-1α has been determined to play vital roles in certain ischemic diseases, such as ischemic stroke, liver, kidney ischemia/reperfusion injury [66–68], and retinal ischemic injury [69], but the exact mechanisms in acute glaucoma remain unclear. Previous studies have demonstrated that HIF-1α can mediate NLRP3 inflammasome activation and IL-1β secretion [70–72]. In additiong, the NLRP3 inflammasome can be activated via CASP8 stimulation [73]. We therefore hypothesized that there may be a regulatory link between CASP8 and HIF-1α. First, we validated the triggering effects of HIF-1α and CASP8 on pyroptosis in vivo and in vitro, respectively. Second, we identified a novel role of CASP8 in inducing HIF-1α signaling via NF-kB translocation: CASP8 promoted NF-kB translocation to drive HIF-1α signaling and thus exacerbate pyroptosis and retinal damage. To elucidate the downstream molecular pathway of CASP8-HIF-1α signaling, we employed selective knockdown strategies. CASP8-HIF-1α signaling inhibition is associated with reduced NLRP12/NLRP3/NLRC4 activation, suggesting that CASP8-HIF-1α signaling is the upstream regulator of NLRP3/NLRP12/NLRC4 in high IOP-induced retinal ischemic injury. Together, these findings demonstrate that CASP8 induces the HIF-1α-NLRP12/NLRP3/NLRC4-IL-1β axis, exacerbating retinal ischemic neuroinflammation.

IL-1β is an important inflammatory mediator of the CNS [74]. In the current study, we explore the functions of IL-1β in mediating neuroinflammation during elevated IOP-induced retinal ischemic injury in acute glaucoma. Our findings revealed novel reciprocal interactions between mature IL-1β and NLRP12/NLRP3/NLRC4, suggesting that the NLR-dependent maturation of IL-1β positively enhances the activation of multiple proinflammatory NLRs to amplify the inflammatory cascades. We also found that IL-1β boosts CASP8 and HIF-1α activation, implicating IL-1β as a crucial mediator that amplifies the neuroinflammatory cascades in elevated IOP-induced retinal ischemic injury by driving CASP8-HIF-1α signaling and subsequently activating various NLRs. It has been demonstrated that GSDMD controls IL-1β secretion [75, 76], and our study validates a new role for IL-1β as a promoter of GSDMD cleavage to induce pyroptosis, suggesting that IL-1β aggravates pyroptosis during high IOP-induced retinal ischemic injury. Given these novel findings, we have determined that IL-1β is a central factor enhancing CASP8-HIF-1α signaling to subsequently increase NLRP3/NLRP12/NLRC4 activation and to initiate pyroptosis during retinal ischemia.

## Conclusions

Our study has revealed the novel mechanisms of pyroptosis and neuroinflammation associated with irreversible RGCs death and vision loss in acute glaucoma. We have demonstrated that genetic deletion of GSDMD reduces RGCs death and the severity of retinal tissue injury. Remarkably, our findings reveal for the first time reveal, that CASP8 can promote HIF-1α signaling via NF-kB translocation. Moreover, NLRP12 coordinates with NLRP3 and NLRC4 to induce IL-1β processing and pyroptotic cell death in a manner dependent on the CASP8-HIF-1α pathway, and genetic deletion of CASP8 depresses pyroptosis dependent on CASP1-induced GSDMD cleavage. Furthermore, mature IL-1β initiates the neuroinflammation cascades and pyroptotic reaction by promoting the CASP8-HIF-1α-NLRP12/NLRP3/NLRC4 pathway and GSDMD cleavage. This process provides a positive feedback circuit which is likely to magnify the inflammatory cascades during retinal ischemic injury. Our study provides novel insights into the roles of pyroptosis and neuroinflammation in elevated IOP-induced retinal ischemic injury and suggests that targeting constraint of CASP8-HIF-1α-NLRP12/NLRP3/NLRC4-initiated neuroinflammation and pyroptosis may be a promising strategy for innovative treatment of acute glaucoma.

## Supplementary information

**Additional file 1 **Supplementary Figure 1. Suppressing NLRP12/NLRP3/NLRC4 significantly improves RGCs survival. **a** Representative immunofluorescence images of retinas from WT and NLRP12 KO mice subjected to RIR injury (*n* = 6). RBPMS was used to identify RGCs (red). Scale bar: 100 μm. **b** Representative immunofluorescence images of retinas from mice subjected to RIR injury (*n* = 6). RBPMS was used to identify RGCs (red). Scale bar: 100 μm. WT: wide type; KO: knockout; RIR: retinal ischemia-reperfusion; GCL: ganglion cell layer; INL: inner nuclear layer; ONL: outer nuclear layer; si: siRNA. The data shown are representative of at least three independent experiments.

**Additional file 2 **Supplementary Figure 2. The expression of NLRP3/ NLRP12/ NLRC4 and GSDMD in primary microglia. **a** Representative immunofluorescence images of murine primary microglia, identified by the microglial-specific marker IBA-1 (*n* = 6). Scale bar: 100 μm. **b**-**e** Representative immunofluorescence images of expression of NLRP3 /NLRP12/ NLRC4 and GSDMD in murine primary microglia exposed to OGDR treatment (*n* = 6). Scale bar: 100 μm. The data shown are representative of at least three independent experiments.

## Data Availability

The datasets used and/or analyzed during the current study are available from the corresponding author on reasonable request.

## Abbreviations

ARRIVE: Animal Research: Reporting of In Vivo Experiments
CASP8: Caspase-8
CASP1: Caspase-1
CNS: Central nervous system
DAMP: Danger-associated molecular pattern
DMEM: Dulbecco’s Modified Eagle’s Medium
FG: Fluoro-gold
FBS: Fetal bovine serum
GSDMD: Gasdermin D
HIF-1α: Hypoxia activates hypoxia-inducible factor-1α
H & E: Hematoxylin and eosin staining
IOP: Intraocular pressure
LPS: Lipopolysaccharide
NLR: Nucleotide-binding leucine-rich repeat containing receptor
NLRC4: NLR family CARD domain-containing protein 4
NLRP3: NLR family pyrin domain-containing 3
NLRP12: NLR family pyrin domain-containing 12;
OGDR: Oxygen and glucose deprivation/reoxygenation
PVDF: Polyvinylidene difluoride
PFA: Paraformaldehyde
RIR: Retinal ischemia-reperfusion
RGCs: Retinal ganglion cells
SEM: Scanning electron microscope
TLR4: Toll-like receptor 4
