# Supplementary figures and images for: NLRP12 collaborates with NLRP3 and NLRC4 to promote pyroptosis inducing ganglion cell death of acute glaucoma
Source: Mol Neurodegener. 2020 Apr 15;15:26. doi: 10.1186/s13024-020-00372-w (PMC7161290; doi:10.1186/s13024-020-00372-w)

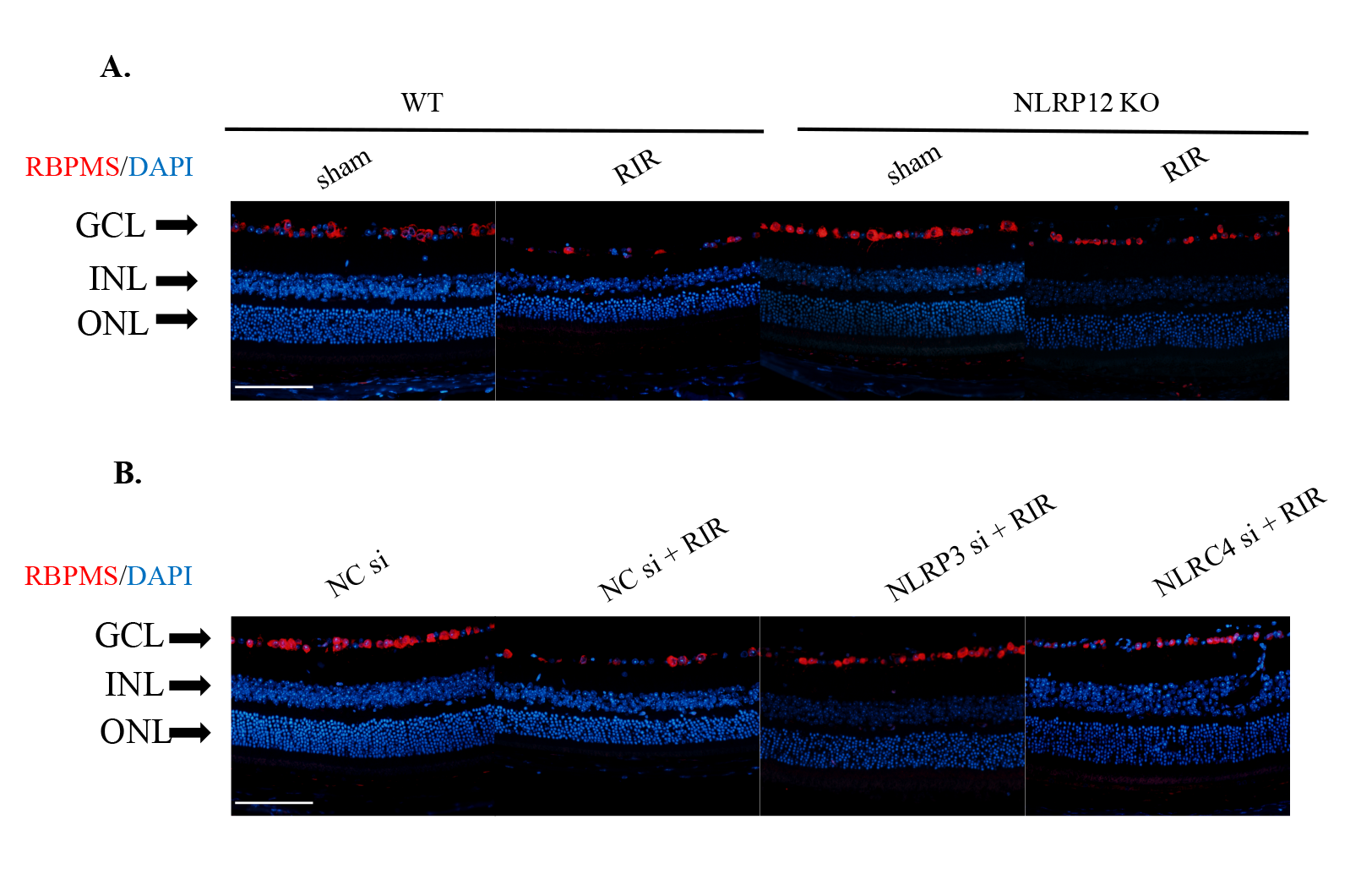

Supplement: Supplementary file 1 — Additional file 1 Supplementary Figure 1. Suppressing NLRP12/NLRP3/NLRC4 significantly improves RGCs survival. a Representative immunofluorescence images of retinas from WT and NLRP12 KO mice subjected to RIR injury (n = 6). RBPMS was used to identify RGCs (red). Scale bar: 100 μm. b Representative immunofluorescence images of retinas from mice subjected to RIR injury (n = 6). RBPMS was used to identify RGCs (red). Scale bar: 100 μm. WT: wide type; KO: knockout; RIR: retinal ischemia-reperfusion; GCL: ganglion cell layer; INL: inner nuclear layer; ONL: outer nuclear layer; si: siRNA. The data shown are representative of at least three independent experiments. [file 13024_2020_372_MOESM1_ESM.tif]

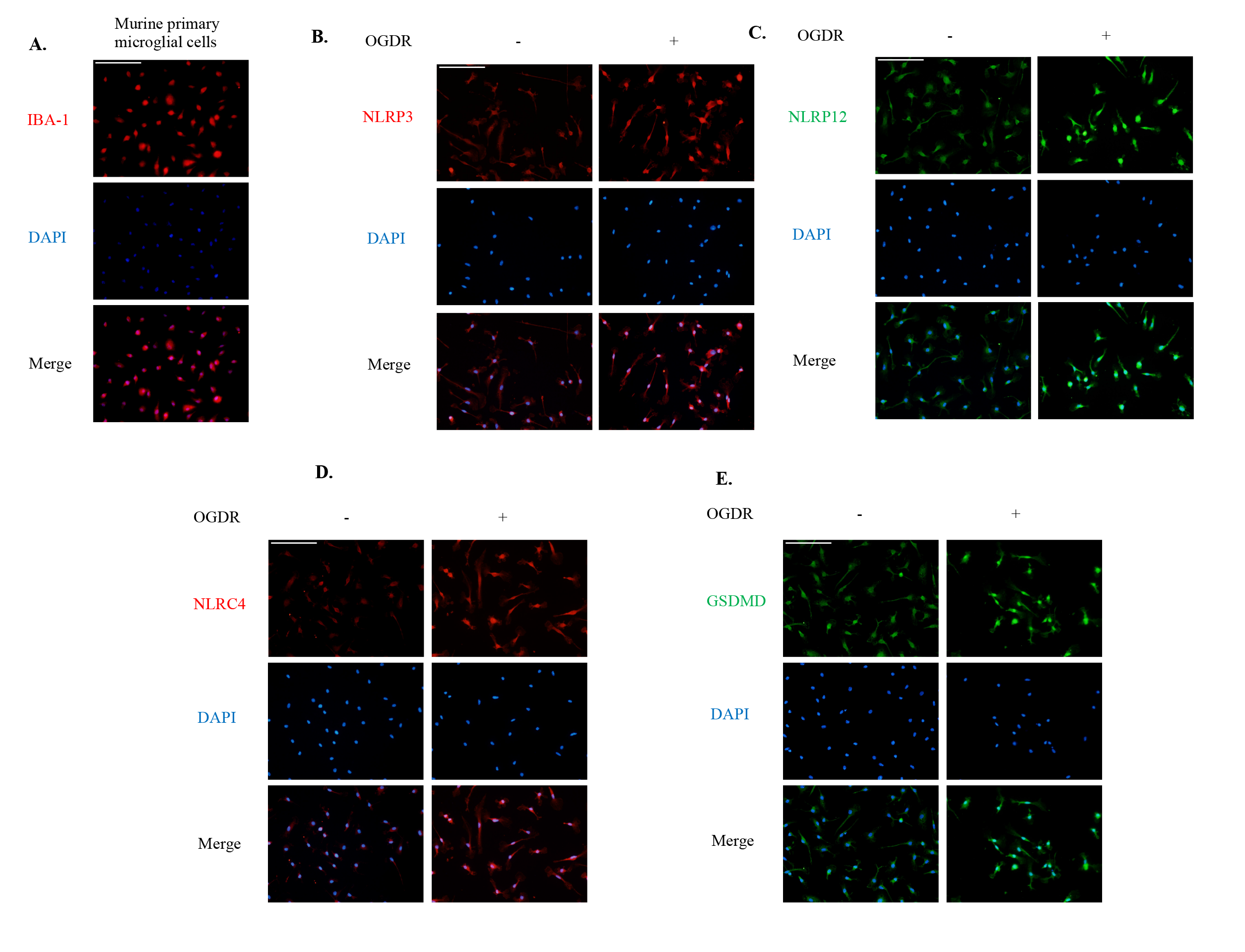

Supplement: Supplementary file 2 — Additional file 2 Supplementary Figure 2. The expression of NLRP3/ NLRP12/ NLRC4 and GSDMD in primary microglia. a Representative immunofluorescence images of murine primary microglia, identified by the microglial-specific marker IBA-1 (n = 6). Scale bar: 100 μm. b-e Representative immunofluorescence images of expression of NLRP3 /NLRP12/ NLRC4 and GSDMD in murine primary microglia exposed to OGDR treatment (n = 6). Scale bar: 100 μm. The data shown are representative of at least three independent experiments. [file 13024_2020_372_MOESM2_ESM.tif]
